# The Rice *TCM5* Gene Encoding a Novel Deg Protease Protein is Essential for Chloroplast Development under High Temperatures

**DOI:** 10.1186/s12284-016-0086-5

**Published:** 2016-03-21

**Authors:** Kailun Zheng, Jian Zhao, Dongzhi Lin, Jiaying Chen, Jianlong Xu, Hua Zhou, Sheng Teng, Yanjun Dong

**Affiliations:** Development Center of Plant Germplasm Resources, College of Life and Environmental Sciences, Shanghai Normal University, Shanghai, 200234 China; Shanghai Institute for Biological Sciences, Chinese Academy of Sciences, Shanghai, 200032 China; Institute of Crop Sciences, Chinese Academy of Agricultural Sciences, 12 South Zhong-Guan Cun Street, Beijing, 100081 China; Present address: Agricultural Faculty, Hokkaido University, Sappro, 060-0817 Japan

**Keywords:** Chloroplast development, Deg protease, Photosystem, Rice

## Abstract

**Background:**

High temperature affects a broad spectrum of cellular components and metabolism in plants. The Deg/HtrA family of ATP-independent serine endopeptidases is present in nearly all organisms. Deg proteases are required for the survival of *Escherichia coli* at high temperatures. However, it is still unclear whether rice Deg proteases are required for chloroplast development under high temperatures.

**Results:**

In this study, we reported the first rice *deg* mutant *tcm5* (*thermo-sensitive chlorophyll-deficient mutant 5*) that has an albino phenotype, defective chloroplasts and could not survive after the 4–5 leaf seedling stage when grown at high temperature (32 °C). However, when grown at low temperatures (20 °C), *tcm5* has a normal phenotype. Map-based cloning showed that *TCM5* encoding a chloroplast-targeted Deg protease protein. The *TCM5* transcripts were highly expressed in all green tissues and undetectable in other tissues, showing the tissue-specific expression. In *tcm5* mutants grown at high temperatures, the transcript levels of certain genes associated with chloroplast development especially PSII-associated genes were severely affected, but recovered to normal levels at low temperatures. These results showed important role of *TCM5* for chloroplast development under high temperatures.

**Conclusions:**

The *TCM5* encodes chloroplast-targeted Deg protease protein which is important for chloroplast development and the maintenance of PSII function and its disruption would lead to a defective chloroplast and affected expression levels of genes associated with chloroplast development and photosynthesis at early rice seedling stage under high temperatures.

**Electronic supplementary material:**

The online version of this article (doi:10.1186/s12284-016-0086-5) contains supplementary material, which is available to authorized users.

## Background

Chloroplast is a semi-autonomous organelle and carries out photosynthesis: the capture of light energy and its conversion into chemical energy. It is known that chloroplast development consists of a series of complex events related to chloroplast differentiation and can be divided into three steps which are coordinately regulated by plastid and nucleus genes (Jarvis and López-Juez 2013; Kusumi et al. 2011; Kusumi and Iba, 2014). The first step involves the activation of plastid replication and plastid DNA synthesis. The second step is the chloroplast ‘build-up’, characterized by the establishment of chloroplast genetic system. At this step nuclear-encoded plastid RNA polymerase (NEP) preferentially transcribes plastid genes encoding plastid gene expression machineries (Hajdukiewicz et al. 1997), and the transcription/translation activities in the chloroplast are dramatically increased. At third step, the plastid and nuclear genes encoding photosynthetic apparatus are expressed at very high levels. Plastid genes for photosynthetic apparatus are mainly transcribed by plastid-encoded RNA polymerase (PEP) (Santis-Maciossek et al. 1999). All expressions of these genes lead to the synthesis and assembly of photosynthetic machineries. In spite of these, the mechanisms of the major genes in higher plants remain largely unknown (Pfalz and Pfannschmidt 2013).

The family of Deg proteases (for degradation of periplasmic proteins) (Strauch and Beckwith 1988), also named as HtrA proteases (for high temperature requirement A) (Lipinska et al. 1988), is one important group among proteolytic enzymes. The Deg proteases, consisting of three representative proteins (DegP, DegQ and DegS), were initially found in *Escherichia coli* and well-studied in *Escherichia coli* and mammals. Most Deg members contain one to four PDZ protein-protein interaction domains (Clausen et al. 2002). A first *E.coli* DegP was identified based on the fact that it is required for the survival at high temperatures (Lipinska et al. 1988, 1990; Strauch and Beckwith 1988). A second *E.coli* protease, DegS, acts in a stress signaling cascade sensing misfolded proteins in the periplasm and transducing the signal to the cytoplasm (Walsh et al. 2003). A third *E.coli* protease, DegQ, is a periplasmic protease but also shares some functional features with DegP (Kolmar et al. 1996). However, relatively little is known about members of this family in plants (Kato and Sakamoto, 2010). To date, although it was predicated to contain 16 genes encoding putative Deg proteases in *Arabidopsis thaliana* (Huesgen et al. 2005), 15 in *Oryza sativa* (Tripathi and Sowdhamini 2006) and 20 in *Populus trichocarpa* (Garcia-Lorenzo et al. 2006), but barely a few Deg proteases from Arabidopsis have been well-studied. Six AtDeg proteases are located in chloroplasts (Itzhaki et al. 1998), one in peroxisomes (Schuhmann et al. 2008), one in mitochondria (for review, see Schuhmann et al. 2012), and one in the nucleus (Pendle et al. 2005). AtDeg3 and AtDeg10 were reported to locate in both chloroplast and mitochondrion, while AtDeg7 is located in both nucleus and mitochondrion (Tanz et al. 2014). Additionally, the Deg proteases were reported to chloroplast-located be involved in the degradation of damaged photosynthetic proteins, especially the photosystem II (PSII) reaction center D1 protein (Kato et al. 2012). For example, the chloroplast-targeted AtDeg1 acts as a chaperone, assisting in the assembly of PSII dimers and supercomplexes (Sun et al. 2010b). The AtDeg15 is a processing enzyme, cleaving the N-terminal peroxisomal targeting signal 2 (Helm et al. 2007; Schuhmann et al. 2008). Recently, it was precisely predicated to contain 10 Deg proteases and 6 DegP-like proteases (Schuhmann et al. 2012) in *Oryza sativa*. However, to our knowledge, any *deg* rice mutants have not been reported yet, still less the function.

Here we report a detailed phenotypic analysis of the rice *deg* mutant, *tcm5*, which exhibited the albino phenotype before the 3-leaf stage at 32 °C, whereas normal green at 20 °C, and the cloning of *TCM5*, encoding a Deg protease protein. Additionally, the transcripts of certain genes for chlorophyll biosynthesis, photosynthesis and chloroplast development were severely affected in *tcm5* mutants at high temperatures. Our work implicates that rice *TCM5* plays an important role in chloroplast development and maintenance of PSII function under high temperatures.

## Results

### Characterization of the *tcm5* Mutant

**Fig. 1:**
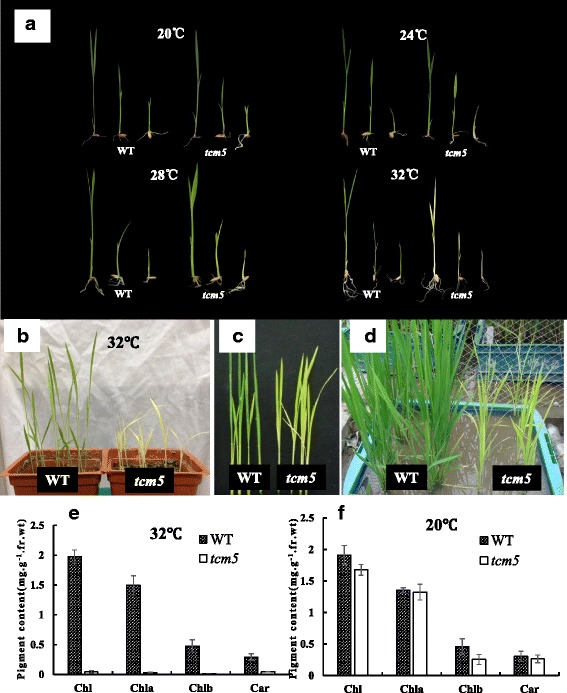
Characterization of the *tcm5* mutants; **a**, 1-, 2-, 3-leaf stage seedlings of wild type (WT) (*left*) and *tcm5* mutants (*right*) grown at 20, 24, 28 and 32 °C, respectively; **b**, 4-leaf-stage plants of wild type (*left*) and *tcm5* mutants (*right*) grown at 32 °C; **c**,**d**, indicate 25-day-old (**c**) and 50-day-old (**d**) plants grown at rice paddy field, respectively (2010, Shanghai, China); **e**, **f**, indicate the pigment contents in the 3^rd^ leaves of WT and mutants grown at 32 and 20 °C, respectively. *Chl* total chlorophyll, *Chla* chlorophyll a, *Chlb* chlorophyll b, *Car* carotenoid, *WT* wide type

The leaves of *tcm5* mutants displayed an albino phenotype (Fig. 1a) before the 3-leaf-stage, and could not survive after the 4–5 leaf stage at 32 °C (Fig. 1b), but they turned yellowish green at 28 °C. Interestingly, the *tcm5* mutants showed no obvious phenotypic differences from the wild type (WT) plants at 20 °C. Similarly, at 32 °C, the Chl a, Chl b and carotenoid (Car) contents in *tcm5* mutants at the 3-leaf stage were drastically lower than those in WT plants (Fig. 1e), however, the pigment levels were similar to WT plants at 20 °C (Fig. 1f). In addition, during the whole growth periods, leaf Chl contents (SPAD values) in *tcm5* plants always showed lower levels than those in WT plants, moreover, the difference ranges become smaller when climate temperatures gradually decreased from summer to autumn in 2010 (Shanghai, China) (Additional file 1: Figure S1). These results indicated that the mutant trait was high-temperature sensitive

PSII complexes are located in the chloroplast. PSII activity of the 3-leaf-stage *tcm5* and WT seedlings at 20 and 32 °C was surveyed by measuring the ratio of variable fluorescence to maximum fluorescence as follows: *Fv/Fm* = (F*m*-F*o*)/F*m*, where *Fo* and *Fm* are minimum and maximum chlorophyll a fluorescence of dark-adapted leaves, respectively. *Fv/Fm* reflects the maximum potential capacity of the PSII photochemical reactions (Krause and Weis 1991). At 32 °C, the *Fv/Fm* was 0.787 ± 0.03 in WT plants and 0.365 ± 0.02 in *tcm5* mutants, indicating that the photochemical efficiency of PSII was significantly suppressed in the *tcm5* mutants. In addition, *ΦPSII* represents the actual photosynthesis efficiency. At 32 °C, the *ΦPS*II value was 0.624 ± 0.04 in WT plants and 0.361 ± 0.04 in *tcm5* mutants, showing that the photosynthesis efficiency was significantly reduced in *tcm5* mutants. By contrast, at 20 °C, all respective values in the *tcm5* mutants were similar to WT levels (Additional file 2: Table S1). Thus, the *tcm5* mutation severely inhibited the PSII activity under high temperatures.

**Fig. 2:**
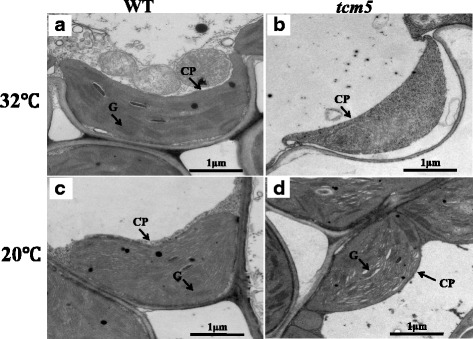
Transmission electron microscopic images of chloroplasts in WT and *tcm5* mutant; **a**, **b**, Chloroplast structure in WT(**a**) and *tcm5* (**b**) cell at 32 °C; **c**, **d**, Chloroplast structure in WT(**c**) and *tcm5*(**d**) cell at 20 °C. *CP, chloroplast; G, grana lamella stacks*

We next examined whether the lack of photosynthetic pigments in *tcm5* mutants was accompanied by ultrastructural changes in the chloroplasts. At 32 and 20 °C, we observed well-developed chloroplasts, with dense and well-structured grana lamella stacks, in WT plants, (Fig. 2a,c). By contrast, at 32 °C we did not observe any well-developed chloroplasts and well-structured grana lamella stacks (Fig. 2b) in *tcm5* mutants. However, there was no obvious difference between *tcm5* and WT plants at 20 °C (Fig. 2d). These results indicated that the *tcm5* mutation causes abnormal chloroplast development at high temperatures.

We also surveyed some agronomic characters of the *tcm5* and WT plants and found that, under field condition, the grain and panicle number per plant (Additional file 1: Figure S2) was significantly decreased, showing that the mutation inhibited the plant growth in *tcm5* mutants.

### Map-based Cloning of *TCM5*

**Fig. 3:**
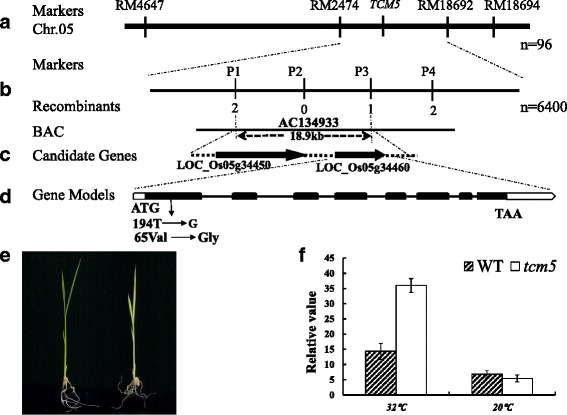
Genetic analysis and cloning of the *TCM5* gene; **a**, The *TCM5* gene was initially located between the RM2474 and RM18692 SSR markers on chromosome 5 using 96 F_2_ mutant individuals; **b**, *TCM5* was narrowed to a 18.9 kb between InDel marker P1 and CAPS marker P3 and co-segregated with the CAPS marker P2 using 6400 F_2_ mutant individuals; **c**, The target region contains two predicted candidate genes (*LOC_Os05g34450*, *LOC_Os05g34460*); **d**, A single nucleotide mutation at codon 194(T→G) in the first exon in *LOC_Os05g34460*, consisting of eight exons; **e**, The complemented plants with pCAMBIA1301:CaMV35S:TCM5 (*left*) and transgenic T_0_ plants with empty vector pCAMBIA1301(*right*); **f**, Transcript levels of *TCM5* in WT and *tcm5* at the 3-leaf stage grown at 32 and 20 °C, *OsActin* was used as a control for qPCR

We used map-based cloning to identify the *TCM5* locus. To this end, we isolated 96 individuals with the albino phenotype from the segregating 412 F_2_ plants derived from the cross of Pei’ai 64S and *tcm5* mutant. The segregation ratio (3:1) of green to albino plants showed that the mutant phenotype is controlled by a single recessive gene (*tcm5*) (Additional file 2: Table S2). First, by genotyping the 96 mutant individuals, we localized *tcm5* between RM2474 and RM18692 on chromosome 5 (Fig. 3a). To fine-map *TCM5* gene, we developed new InDel and CAPS markers between RM2474 and RM18692 (Additional file 2: Table S3). The *TCM5* locus was finally narrowed to an 18.9-kb between P1 and P3 markers using 6400 F_2_ mutant individuals (Fig. 3b). The target region was predicated to contain two candidate genes (*LOC_Os05g34450*, *LOC_Os05g34460*) (Fig. 3c). We sequenced both candidate genes and only found a single nucleotide mutation (T→G) at position 194 bp from the ATG start codon in *LOC_Os05g34460*, which resulted in an amino acid change from Val to Gly (Fig. 3d).

To further prove that the lack of *TCM5* was responsible for the mutant phenotype in the *tcm5* mutants, genetic complementation was performed. Resultantly, all of the 11 transgenic plants with pCAMBIA1301:CaMV35S:TCM5 were obtained and displayed wild-type phenotype under high temperatures (Fig. 3e). Meanwhile, 13 independent lines were transformed with empty vector, pCAMBIA1301, all failed to rescue the *tcm5* mutant. These results confirmed that *LOC_Os05g34460* is *TCM5*.

### Characterization of TCM5

The *TCM5* encoded a putative Deg protease homologue, also predicated and named as OsDeg10 (Schuhmann et al*.*^2012^). Searching the rice genome database revealed that *TCM5* is a single-copy gene. The *TCM5* gene is comprised of eight exons and encodes a 614-amino acid protein with the molecular mass of approximately 67kD. In addition, TCM5 was predicated to contain not only a chloroplast transit peptide of 94 amino acids at the N terminus, but also two PDZ domains of trypsin-like serine proteases. We found that the *tcm5* mutation site occurred at chloroplast transit peptides.

**Fig. 4:**
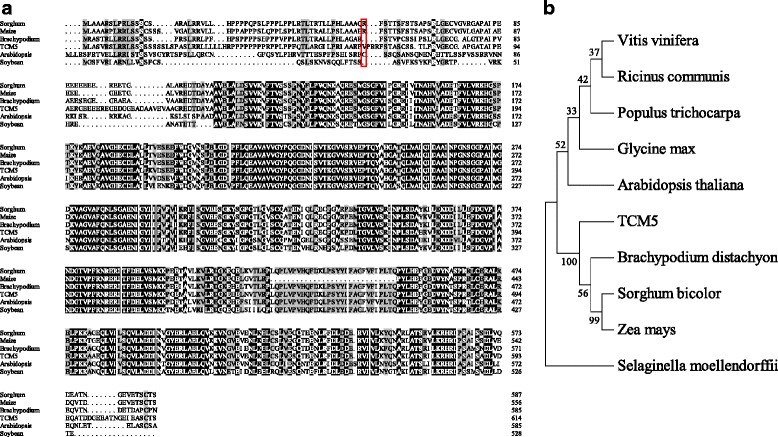
Phylogenic analysis of TCM5; **a**, Amino acid sequence alignment of *TCM5* with the five homologs. Amino acids fully or partially conserved are *shaded black* and *gray*, respectively. In the *red box* is the amino acid substitution in the *tcm5* mutant; **b**, Phylogenic tree of TCM5 and homologs. Protein sequences are *Ricinus communis* (RCOM_1076350), *Populus trichocarpa* (POPTR_0008s07940), *Arabidopsis thaliana* (AT5G36950, AtDeg10), *Glycine max* (100802340), *Vitis vinifera* (100250366), *Brachypodium distachyon (*Bradi2g25260), *Sorghum bicolor* (Sb09g020480), *Zea mays* (GRMZM2G117615), *Selaginella moellendorffii* (SELMODRAFT_62730). The rooted tree is based on a multiple sequence alignment generated with the program Mega6. Scale represents percentage substitution per site. Statistical support for the nodes is indicated

BLAST searches found that there are 10 Deg protease and 6 Deg-like protease proteins in the whole rice genome as described by Schuhmann et al.(2012) and TCM5 shares the highest sequence similarity to LOC_Os02g50880 (OsDeg9.1) (52.6 % amino acid identity) (Additional file 1: Figure S3). Additionally, close homology of TCM5 were also indented in *Arabidopsis thaliana*, *Brachypodium distachyon*, *Glycine max*, *Sorghum bicolor*, and *Zea mays*, showing the high conservative property within higher plants. And, Bradi2g25260 from *Brachypodium distachyon* shares the highest homology to TCM5 (88.1 % amino acid identity) (Fig. 4a). However, the functions of these homologs remain unclear. As shown in Fig. 4b, TCM5 homologs can be clearly divided into two groups: monocots and dicotyledons, consistent with the biological taxonomy (Fig. 4b).

### *TCM5* Expression

**Fig. 5:**
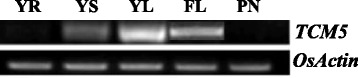
Expression analysis of *TCM5* by RT-PCR analysis; YR, young-seedling roots; YS, young-seedling stem; YL, young-seedling leaf; FL, flag leaf at heading; PN, young panicles. *OsActin* was used as a control (cycle number for *OsActin* was 28, cycle number for *TCM5* was 35)

To examine the expression pattern of *TCM5*, we used RT-PCR to investigate the transcript levels of *TCM5* in various tissues (Fig. 5). Resultantly, *TCM5* was highly expressed in all green tissues (leaf and stem) and undetectable in roots and panicles, showing its tissue-specific expression.

To further explore whether the *tcm5* mutation and temperature influence *TCM5* expression, we examined *TCM5* transcript levels in 3-leaf *tcm5* and WT seedlings grown at 20 and 32 °C (Fig. 3f). Interestingly, regardless of WT and *tcm5* plants*, TCM5* transcript levels were more strongly expressed at high temperatures than those at low temperatures. Specifically, at 32 °C, *TCM5* transcript levels in the *tcm5* mutants were over 2-fold higher than WT levels. However, at 20 °C, *TCM5* expression levels were relatively low and comparable to WT plants (Fig. 3f). These results suggested that *TCM5* function is more needed for chloroplast development under high temperatures.

### Subcellular Localization of TCM5 Protein

**Fig. 6:**
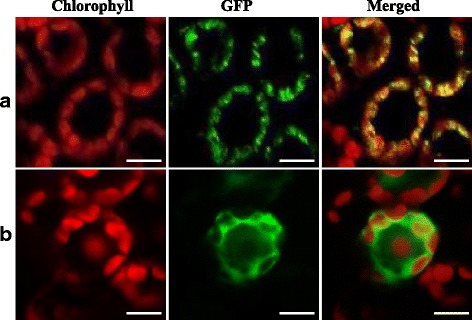
Subcellular localization of TCM5 protein; **a**, TCM5-GFP fusion; **b**, Empty GFP vector without a specific targeting sequence. The scale bar represents 20 μm

The TCM5 protein was predicted to localize to chloroplasts by TargetP (Emanuelsson et al. 2000, http://www.cbs.dtu.dk/services/TargetP/). To investigate the actual subcellular localization of TCM5, the cDNA fragment encoding the N-terminal region (amino acids 1–135) of TCM5 was introduced into the N-terminal of the GFP gene in the expression vector pMON530-GFP. Then we transformed the pMON530:TCM5-GFP plasmids into tobacco cells by Agrobacterium-mediated infection, and used empty pMON530-GFP vector without a specific targeting sequence as the positive control. Confocal laser scanning microscopy was used to observe the fluorescent signals after 48 h transformation. The green fluorescent signal of the TCM5-GFP fusion protein perfectly co-localized with the chlorophyll autofluorescence (Fig. 6a) in tobacco cells. By contrast, the cells transformed with the empty GFP vector had green fluorescent signals in both the cytoplasm and the nucleus (Fig. 6b). These findings confirmed that TCM5 was localized to the chloroplast.

### The Transcript Expressions of Functionally Related Genes in the *tcm5* Mutants

**Fig. 7:**
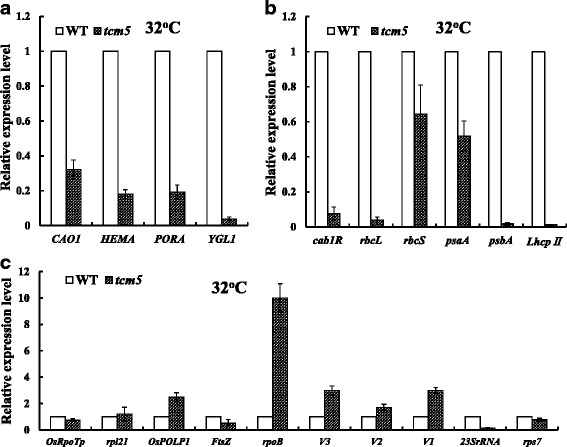
qPCR analysis of those genes associated with Chl biosynthesis, photosynthesis and chloroplast development at 32 °C; **a**, **b**, **c** Expression levels of genes related to Chl biosynthesis (**a**), photosynthesis (**b**) and chloroplast development (**c**) in WT and *tcm5* mutant in the 3^rd^ leaves, respectively. The relative expression level of each gene in WT and mutant was analyzed by qPCR and normalized using the *OsActin* as an internal control. Data are means ± SD (*n* = 3)

We examined the transcript levels of genes associated with Chl biosynthesis, photosynthesis and chloroplast development (*PORA, HEMA, CAO1, YGL1, cab1R, rbcL, rbcS, psaA, psbA, LhcpII,V1, V2, V3, OsRpoTp, OsPoLp1, rpoB, 23SrRNA, rps7, rps21, FtsZ*) in the *tcm5* mutants. Under high temperatures, the transcript levels of all tested genes for Chl biosynthesis (Fig. 7a), such as glutamyl tRNA reductase (*HEMA*) and chlorophyllide a oxygenase (*CAO1*), NADPH:protochlorophyllide oxidoreductase (*PORA*) and Chl synthase(*YGL1*), were obviously reduced in *tcm5* mutants, compared with WT plants, consistent with the decreased of Chl content (Fig. 1e) and the albino phenotype (Fig. 1a). We also investigated the photosynthesis-related genes (Fig. 7b) *cab1R*, encoding the Chl a/b binding protein, *rbcL*, encoding the large subunit of ribulose-1,5-bisphosphate carboxylase (Rubisco), *rbcS*, encoding the small subunit of Rubisco, *psaA* and *psbA* for the reaction center polypeptides in photosystems and *LhcpII* encoding light harvesting complex protein in PS II, and found that all these genes were obviously suppressed in *tcm5* mutants especially PSII-associated *psbA* and *LhcpII*, consistent with the low photochemical efficiency and photosynthesis efficiency in *tcm5* mutants under high temperatures (Additional file 2: Table S1).

**Fig. 8:**
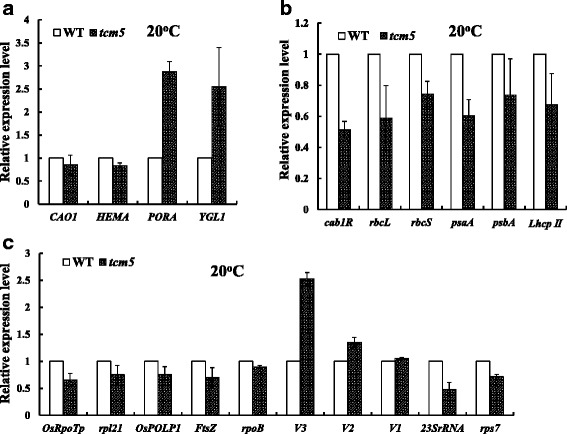
qPCR analysis of those genes associated with Chl biosynthesis, photosynthesis and chloroplast development at 20 °C. **a**, **b**, **c** Expression levels of genes related to Chl biosynthesis (**a**), photosynthesis (**b**) and chloroplast development (**c**) in WT and *tcm5* mutants in the 3^rd^ leaves, respectively. The relative expression level of each gene in WT and mutant was analyzed by qPCR and normalized using the *OsActin* as an internal control. Data are means ± SD (*n* = 3)

For chloroplast development-associated transcripts, we investigated the expressions of *V3 (RNRL)* encoding the large subunits of ribonucleotide reductase (Yoo et al. 2009), *V2*, encoding plastid/mitochondrial guanylate kinase (pt/mt GK) (Sugimoto et al. 2007), *V1(NUS1)*, encoding protein NUS1 (Kusumi et al. 2011), *OsRpoTp*, encoding NEP core subunits (Hiratsuka et al. 1989), and *rpoB* encoding one PEP core subunit (Inada et al. 1996; Kusumi et al. 2011) and *OsPoLP1*, encoding one plastidal DNA polymerase (Takeuchi et al. 2007), and *FtsZ*, encoding a component of the plastid division machinery (Vitha et al*.*^2001^). Also we examined the transcript levels of ribosome-associated genes, *23S rRNA* (23S ribosomal RNA), encoding one component of the plastid translation machinery, and *rpl21* and *rps7*, encoding the large and small subunits ribosomal protein, respectively. Resultantly, with the exception of the not significant effects on *FtsZ*, *OsRpoTp*, *rps7* and *rpl2* in the *tcm5* mutants grown at 32 °C, other genes (*rpoB*, *V3*, *V2*, *V1*, *OsPoLP1,* and *23S rRNA*) were significantly affected (Fig. 7c), resulting in abnormal chloroplast (Fig. 2b). Overall, the *tcm5* mutation affected the transcriptional levels of some genes associated with not only Chl biosynthesis, photosynthesis but also the chloroplast development under high temperatures. By contrast, in the *tcm5* mutants grown at 20 °C, all transcripts of the affected genes at 32 °C (Fig. 8) were, at least, partly recovered to WT levels. Accordingly, the differentially expressed levels of so many key genes mentioned above under low or high temperatures might lead to the thermo-sensitivity for leaf-color in the *tcm5* mutant.

## Discussion

In this study, we reported a rice nuclear-encoded Deg proteases protein *TCM5*, which is required for chloroplast development under high temperatures. Its disruption led to abnormal chloroplast development and albino phenotype of rice at high temperatures, resulting from abnormally expressed transcripts of those genes associated with chlorophyll biosynthesis, photosynthesis and chloroplast development. Our work demonstrated that *TCM5* plays an important role in chloroplast development under high temperatures.

### *TCM5* is Essential for Chloroplast Development under High Temperatures

The chloroplast is a semi-autonomous organelle, which contains about 100 genes, more than 3000 proteins function within it (Leister 2003). Thus, tightly coordinated gene expression between the plastid and nuclear genomes is essential for chloroplast development. The mutation of those genes could result in chlorophyll deficient/chloroplast defects in plants. At present, over 70 chlorophyll/chloroplast deficient mutants in rice have been identified (for review, see Kurata et al. 2005). Among them *v1*, *v2*, *v3*, *v5*, *v7*, *chs1*, *chs2*, *chs3*, *chs4*, *and chs5* mutants have been identified to be low temperature sensitive (Kusumi and Iba 2014). Recently, two genes, *TCD9* encoding α subunit of chaperonin protein 60 (Jiang et al. 2014) and *OsV4* encoding a PPR protein (Gong et al. 2014), essential for chloroplast development at low temperature were identified in rice. In contrast, six high-temperature sensitive rice mutants were reported, of which five mutants (Yatou and Cheng 1989) exhibited the chlorotic type over 35 °C, and *cde1(t)* mutant (Liu et al. 2007) displayed the chlorotic type over 26 °C, most likely resulted from the mutation of *OsGluRS*. In this study, the *tcm5* mutants displayed the abnormal chloroplast and even could not survive after the 4–5 leaf stage at 32 °C, but are normal under 20 °C. Judging from these results, there exists different shift sensitive-temperatures and molecular bases for chloroplast development under high temperatures in rice. In addition, the impaired chloroplasts (Fig. 2b) and the abnormal transcripts of genes for chloroplast development (Fig. 7) were observed in *tcm5* mutants at high temperatures, and those affected genes under high temperatures, at least, in part could recover to the comparable levels to WT plants at low temperatures (Fig. 8). These results can explain the changes of chloroplast development and leaf color between 20 and 32 °C (Figs. 1 and 2). Additionally, the highly induced mRNA accumulation (Fig. 3f) of *TCM5* at high temperatures showed that *TCM5* was temperature-regulated gene, consistent with the previous results that Deg proteases are required for the survival of *Escherichia coli* at high temperatures (Lipinska et al. 1988, 1990; Strauch and Beckwith 1988). Our study showed that the *TCM5* was constitutively expressed with high levels of transcripts in all green tissues (Fig. 5) and TCM5 protein distributes throughout the entire chloroplast (Fig. 6a), showing the functions of TCM5 in chloroplast. Also, the lethality of *tcm5* mutants may be due to the block of chloroplast development under high temperatures (Fig. 1b). These results strongly confirm the notion that rice *TCM5* is needed for chloroplast development under high temperatures. However, the reasons why the abnormal chloroplast/albino phenotypes of rice, such as *tcm5* mutant in this study and *cde1(t)* mutant, occur under solely higher temperatures have not been well documented (Yatou and Cheng 1989; Liu et al. 2007). A possible explanation in this study is that the *TCM5* function possibly is not prerequisite under lower temperatures, but is essential under higher temperatures for chloroplast development. This explanation was strongly supported by the results from transcriptional analysis (Fig. 3f) that (a): all low expressions and the existence of no discriminated difference at 20 °C regardless of WT and *tcm5* mutant; (b): high expressions at 32 °C regardless of WT and *tcm5* mutant despite of the higher level in the mutant caused by feedback effects.

As stated above, the process of the chloroplast development from proplastids to mature chloroplasts can be divided into three steps (Jarvis and López-Juez 2013; Kusumi et al. 1997; Kusumi and Iba, 2014). The functions of *OsPOLP1*, *FtsZ* and *V3(RNRL)* encoding plastidal DNA polymerase, a component of plastid division machinery and the large subunits of ribonucleotide reductase respectively (Vitha et al. 2001; Takeuchi et al. 2007; Yoo et al. 2009), are known to involve in the first step. In addition, *OsRpoTp*, *V2*, and *rpoB*, encoding NEP, plastidal guanylate kinase, and PEP β core subunit, respectively (Hiratsuka et al. 1989; Kusumi et al. 2004; Sugimoto et al. 2007) are involved in the second step. In view of not significant decreases of transcripts of key genes mentioned above (Fig. 7), *TCM5* could not function in the first and second steps. In addition, the transcripts of all PEP-transcribed plastid genes (*cab1R*, *rbcL*, *psaA*, *psbA*, *LhcpII*) and nuclear gene *rbcS* involved in the third step were severely suppressed under high temperatures in *tcm5* mutants (Fig. 7), showing that *TCM5* functions the third step. Also, the significantly decreased accumulation of *23S rRNA* (Fig. 7c) in *tcm5* mutants under high temperatures indicated that *TCM5* affects the plastid translation machinery in chloroplasts. These results revealed that *TCM5* is necessary in the process of chloroplast development under high temperatures.

### *TCM5* may be Involved in PSII Function under High Temperatures

The Deg/HtrA family is present in nearly all organisms from bacteria to human and plants. It was known that Deg proteases are essential for the survival of *Escherichia coli* at high temperatures (Lipinska et al. 1988, 1990; Strauch and Beckwith 1988). The chloroplast-targeted Deg proteases were reported to be involved in the degradation of damaged photosynthetic proteins, especially the PSII reaction center D1 protein (for review, see Schuhmann et al. 2012, Kato et al. 2012). In Arabidopsis, AtDeg1 acts as a chaperone, assisting in the assembly of PSII dimers and supercomplexes (Sun et al. 2010b). More interestingly, Arabidopsis *deg* mutants *deg5*, *deg7*, and *deg8* all showed decreased growth including chloroplast development, compared with the WT plants under high light, however, have no obvious differences under normal conditions, all AtDeg5, AtDeg7 and AtDeg8 participate in the repair of PSII after photo inhibition, and *AtDeg5*, *AtDeg7*, and *AtDeg8* were all high-light-dependent genes (Sun et al. 2010). Regrettably, any Arabidopsis *deg10* mutant whose AtDeg10 shares the highest homology to TCM5 (74.7 % amino acid identity, Additional file 1: Figure S4) has not reported yet, still less the function. Judging from the recovered normal chloroplast at low temperatures (<24 °C) in *tcm5* mutants, it could be concluded that *TCM5* is functionally required under high temperatures. The significant decreases of *Fv/Fm*, reflecting the maximum potential capacity of the PSII photochemical reactions, and *ΦPSII*, representing the actual photosynthesis efficiency, in *tcm5* mutants at 32 °C and the similar degrees as WT plants at 20 °C showed that the malfunction of TCM5 barely affected the photosynthesis efficiency under high temperatures. In addition to the similar levels as WT plants at low temperatures (Fig. 8), the transcripts of *psbA* (>50-fold) and *LhcpII* (>80-fold), both encoding the PSII core proteins (D1, Lhcp II), were more significantly suppressed compared to those of *psaA* (<2-fold) encoding the PSI core protein (PsaA) in *tcm5* mutants at high temperatures (Fig. 7b). Thus, it could be reasonably speculated that the TCM5 disruption maybe hinder the PSII function, ultimately leading to the significantly decreased PSII activities (*Fv/Fm* and *ΦPSII*) under high temperatures (32 °C), which was consistent with the previous results of high correlation between thermotolerance and photosystem II activity (Chen et al. 2014). In addition, Arabidopsis Deg7 (AtDeg7, At3g03380), with the low homology to TCM5 (16 % amino acid identity) (data not shown), involving in the primary cleavage of photodamaged D1, D2, CP47, and CP43 in PSII complexes, was reported to repair the PSII function under high-light conditions (Sun et al. 2010a). Considering that *TCM5* is high-temperature-dependent gene, different from high-light-dependent *AtDeg7* (Sun et al. 2010a), we might speculate that, at least, the mechanisms of temperature response are different among them. In addition, TCM5/OsDeg10 was solely located in thylakoid membrane (Fig. 6), not only different from AtDeg1, 5, 8 (on the stromal side) and, AtDeg2, 7(on the lumenal side), but also AtDeg10 (on both chloroplasts and mitochondrion) (Tanz et al. 2014), which clearly showed the different functions even between OsDeg10/TCM5 and AtDeg10.

Taken together, these results show that the *tcm5* mutation affected the third stage of chloroplast development under high temperatures, and the activation of PSII-associated genes is severely weakened, resulting in the suppression of chloroplast development, Chl-biosynthesis and photosynthesis, finally leading to the death under high temperatures. To our knowledge, this is the first experimental evidence that there exists a unique rice Deg/HtrA protease protein essential for chloroplast development under high temperatures. As for *TCM5*, how to participate in chloroplast development and maintain PSII function under high temperatures is needed in the further study.

## Conclusions

The TCM5 encodes chloroplast-targeted Deg protease protein which is important for chloroplast development and the maintenance of PSII function and its disruption would lead to a defective chloroplast and affected expression levels of genes associated with chloroplast development and photosynthesis of rice plants under high temperatures.

## Methods

### Plant Materials and Growth Conditions

The thermo-sensitive chlorophyll-deficient mutant, *tcm5*, used in this study, was obtained from *japonica* rice variety, Jiahua 1(WT), and induced by ^60^Co gamma-radiation in 2006. The F_2_ genetic mapping population was generated from a cross between Pei’ai 64S (*indica*) and the *tcm5* mutant. Rice plants were cultivated in rice paddy fields in Shanghai (summer-autumn season, temperate climate), China, following conventional methods. For phenotypic characterization, chlorophyll (Chl) and carotenoid content measurement and RNA extraction, the wild-type (WT) and *tcm5* seedlings were grown in growth chambers. In the F_2_ population, thermo-sensitive Chl deficient albino plants were selected for genetic mapping.

### Phenotype Characterization, Chlorophyll (Chl) and Carotenoid Measurements

The rice seeds were grown in the growth chambers with 12 h-dark/12 h-light at four temperatures (20, 24, 28, 32 °C) and the light intensity of 120 μmol of photons m^−2^ s^−1^. The extracts were obtained from 100 mg of the third fresh leaves at the 3-leaf stage seedlings and homogenised in 10 ml of 100 % acetone. Spectrophotometric quantification was carried out in BECKMANCOULTER-DU720. Total Chl, Chla, Chlb and carotenoid contents were determined according to the methods of Arnon (1949) and Alan (1994). In 2010, WT and *tcm5* plants were grown in experimental rice fields of Shanghai Normal University by conventional methods. Leaf chlorophyll SPAD values (Additional file 1: Figure S1) by means of CHLOROPHYLL METER (SPAD-502, Minolta Co., Ltd, Japan), which can provide a simple, quick portable and non-destructive method for estimating leaf Chl content (Peng et al. 1993; Turner and Jund 1991; Dwyer et al. 1991) were measured every week from transplanting (summer) to heading (autumn). Finally, agronomic traits of rice plants (Additional file 1: Figure S2) were measured at maturity.

### Chlorophyll Fluorescence Analysis and Transmission Electron Microscopy

Rice seedlings were grown at 20 °C or 32 °C in growth chambers. Chlorophyll fluorescence analyses for the third leaves at the 3-leaf stage were performed with a PAM-2000 portable chlorophyll fluorometer (MINI-PAM; Walz; http://walz.com). The variables *Fo*, *Fm*, *Fv*, the *Fv/Fm* ratio and *ΦPSII* were measured and calculated according to Meurer et al. (1996) after seedlings were dark-adapted for 20 min. Meanwhile, transverse sections of top leaves were sampled from the 3-leaf-stage seedlings grown at 20 °C or 32 °C. Samples were fixed in 4 % glutaraldehyde buffer, 2.5 % glutaraldehyde and 1 % osmic acid phosphate buffer at 4 °C for 5 h after vacuum. After staining with uranyl acetate, tissues were further dehydrated in an ethanol series and finally embedded in Spurr’s medium prior to ultrathin sectioning. Samples were stained again and examined with a Hitachi-7650 transmission electron microscope.

### Positional Cloning of *TCM5*

For genetic analysis, F_2_ population from the cross between *tcm5* and Pei’ai 64S was used for fine mapping of the *TCM5* locus. We adopted 53 SSR primers based on data in Gramene (http://www.gramene.org). New SSR, InDel and CAPS markers (Additional file 1: Table S3) were developed based on the entire genomic sequences of the *japonica* Nipponbare variety (Goff et al. 2002) and the *indica* variety 9311 (Yu et al. 2002). The candidate genes’ function and full length cDNA were acquired using TIGR (http://rice.plantbiology.msu.edu/cgi-bin/gbrowse/rice/) and KOME (http://cdna01.dna.affrc.go.jp/cDNA/index.html) respectively.

### RT-PCR and Quantitative Real-time PCR

To understand the tissue expression pattern of *TCM5*, we extracted wild type RNA from young roots, young stems, young leaves, flag-leaves and young panicles with TRIzol Reagent (Invitrogen; http://www.invitrogen.com) and DNase I treated RNA using an RNeasy kit (Qiagen; http://www.qiagen.com) following the manufacturer’s instructions. The first-strand cDNA was synthesized with the Revert-Aid first-strand cDNA synthesis kit (Toyobo; http://www.toyobo.co.jp) following the manufacturer’s instructions. *OsActin* were internal controls. The specific primers are listed in Additional file 1: Table S4. RT-PCR analysis was carried out to assess the transcriptional levels.

For transcriptional expression analysis of *TCM5* and 20 genes (*PORA*, *CAO1*, *YGL1*, *cab1R*, *rbcL*, *rbcS*, *psaA*, *psbA*, *Lhcp II*, *V3 (RNRL)*, *V1*, *V2*, *OsRpoTp*, *OsPoLp1*, *rpoB*, *23SrRNA*, *rps7*, *rps21*, *FtsZ*) associated with Chl-biosynthesis, chloroplast development and photosynthesis, total RNA was obtained from the third fresh leaves of WT and *tcm5* seedlings grown at 20 °C or 32 °C. Quantitative real-time PCR amplifications were carried out with ABI7500 Real-Time PCR System (Applied Biosystems; http://www.appliedbiosystems.com), and the relative quantification of gene expression data was analyzed as described by Livak and Schmittgen (2001). The data set was normalized using *OsActin* as a control. The specific primers were listed in Additional file 1: Table S4.

### Complementation Analysis

For complementation analysis, cDNA clone (AK073199) plasmid vector (J0133023K08) (http://cdna01.dna.affrc.go.jp) carrying the full-length *TCM5* (*LOC_Os05g34460*) cDNA sequence, was purchased from National Institute of Agrobiological Sciences of Japan in 2011. Then, the 2426 bp DNA fragment, carrying the full-length CDS (1845 bp) of *TCM5*, was amplified using the primers 5′- CCGGAATTCGTCGACCTCTAT -3′ (*EcoR*I) and 5′-TGGCCGGATCCGGACTGTTT-3′ (*BamH*I). The underlined sequences represent cleavage sites of the enzymes. Then, the resulted DNA fragment was cloned into the pMD18-T vector (TaKaRa). Following sequence verification, the fragments were cloned into the pCAMBIA1301 binary vector (CAMBIA; www.cambia.org.au). Finally, the pCAMBIA1301:CaMV35S:TCM5-cDNA plasmids were transferred into *Agrobacterium tumefaciens* EHA105 and introduced into the *tcm5* mutants by Agrobacterium-mediated transformation (Hiei et al. 1994). In this study, transgenic plants were generated from recovered resistant calli by the procedure of Lu et al. (2001).

### Subcellular Localization of GFP Proteins

To investigate the subcellular localization of TCM5 protein, the cDNA fragment encoding the N-terminal region (amino acids 1–135) of TCM5 was amplified from cDNA clone (AK073199) using the primer pairs (pF 5′- GGGGTACCATGCTCGCCTCCGTCC-3′ (*Kpn*I) and pR 5′- CGGCCATGGCGAGTCCAGCGCCAGCT-3′ (*Nco*I) the underlined sequence represents cleavage sites of enzymes) and ligated into the pMON530-GFP vector, in frame with GFP. The resultant pMON530-TCM5-GFP plasmids were introduced into tobacco (*Nicotiana tabacum*) leaves and co-cultured at 25 °C for 2 days. At the same time, the pMON530-GFP empty carrier was used as a control. Then the GFP fluorescences in tobacco cells were observed using a Zeiss confocal laser scanning microscope (LSM 5 PASCAL; http://www.zeiss.com).

### Sequence and Phylogenetic Analysis

Gene prediction and structure analysis were performed using TIGR (http://rice.plantbiology.msu.edu/cgi-bin/gbrowse/rice/).The chloroplast transit peptides (CTP) was predicted by the ChloroP program (http://www.cbs.dtu.dk/services/TargetP/). The full-length amino acid sequences of the TCM5 and homologs identified via BLAST search were aligned with the MUSCLE tool (Edgar 2004) using the default parameters. The phylogenetic tree was constructed and tested by MEGA6 (http://www.megasoftware.net) based on the neighbor-joining method.

## Additional files

Additional file 1: Figure S1. The change of chlorophyll contents (SPAD values) in *tcm5* and WT plants from the 1st (summer) to 12th weeks (heading date, autumn) after transplanting (2010, Shanghai, China). **Figure S2.** Comparative agronomic characteristics of *tcm5* and wild type of rice plants grown under field condition (2010, Shanghai, China). *PH, plant height; PN, panicle number per plant; GW: 1000-grain weight (g); GN, grains per panicle.* **Figure S3.** Phylogenic analysis of rice Deg proteases inculding 10 Deg proteases and 6 Deg-like proteases proteins (Schuhmann et al., 2012) in the rice genome. **Figure S4.** Phylogenic analysis of TCM5 with all Arabidopsis Deg proteases. (PPTX 182 kb) Additional file 2: Table S1. The fluorescence induction parameters. **Table S2.** Genetic segregation analysis of tcm5 mutants in the F2 population. **Table S3.** The PCR-based molecular markers designed for fine mapping. **Table S4.** Markers designed for RT-PCR. (DOC 72 kb)

## References

[CR1] Alan RW (1994). The spectral determination of chlorophylls a and b, as well as total carotenoids, using various solvents with spectrophotometers of different resolution. J Plant Physiol.

[CR2] Arnon DI (1949). Copper enzymes in isolated chloroplasts. polyphenoloxidase in beta vulgaris. Plant Physiol.

[CR3] Chen K, Sun XY, Amombo E, Zhu Q, Zhao ZG, Liang Chen L, Xu QG, Fu JM (2014). High correlation between thermotolerance and photosystem II activity in tall fescue. Photosynth Res.

[CR4] Clausen T, Southan C, Ehrmann M (2002). The HtrA family of proteases: implications for protein composition and cell fate. Mol Cell.

[CR5] Dwyer LM, Tollenaar M, Houwing L (1991). A nondestructive method to monitor leaf greenness in corn. Can J Plant Sci.

[CR6] Edgar RC (2004). MUSCLE: multiple sequence alignment with high accuracy and high throughput. Nucleic Acids Res.

[CR7] Emanuelsson O, Nielsen H, Brunak S, Heijn GV (2000). Predicting subcellular localization of proteins based on their N-terminal amino acid sequence. J Mol Biol.

[CR8] Garcia-Lorenzo M, Sjödin A, Jansson S, Funk C (2006). Protease gene families in Populus and Arabidopsis. BMC Plant Biol.

[CR9] Goff SA, Ricke D, Lan TH, Presting G, Wang R, Dunn M, Glazebrook J, Sessions A, Oeller P, Varma H, Hadley D, Hutchison D, Martin C, Katagiri F, Lange BM, Moughamer T, Xia Y, Budworth P, Zhong J, Miguel T, Paszkowski U, Zhang S, Colbert M, Sun WL, Chen L, Cooper B, Park S, Wood TC, Mao L, Quail P, Wing R, Dean R, Yu Y, Zharkikh A, Shen R, Sahasrabudhe S, Thomas A, Cannings R, Gutin A, Pruss D, Reid J, Tavtigian S, Mitchell J, Eldredge G, Scholl T, Miller RM, Bhatnagar S, Adey N, Rubano T, Tusneem N, Robinson R, Feldhaus J, Macalma T, Oliphant A, Briggs S (2002). A draft sequence of the rice genome (*Oryza sativa* L. ssp. *japonica*). Science.

[CR10] Gong X, Su Q, Lin D, Jiang Q, Xu J, Zhang J, Teng S, Dong Y (2014). The rice *OsV4* encoding a novel pentatricopeptide repeat protein is required for chloroplast development during the early leaf stage under cold stress. J Integr Plant Biol.

[CR11] Hajdukiewicz PT, Allison LA, Maliga P (1997). The two RNA polymerases encoded by the nuclear and the plastid compartments transcribe distinct groups of genes in tobacco plastids. EMBO J.

[CR12] Helm M, Luck C, Prestele J, Hierl G, Huesgen PF, Fröhlich T, Arnold GJ, Adamska I, Görg A, Lottspeich F (2007). Dual specificities of the glyoxysomal/peroxisomal processing protease Deg15 in higher plants. Proc Natl Acad Sci U S A.

[CR13] Hiei Y, Ohta S, Komari KT (1994). Efficient transformation of rice (*Oryza sativa* L.) mediated by Agrobacterium and sequence analysis of the boundaries of the T-DNA. Plant J.

[CR14] Hiratsuka J, Shimada H, Whittier R, Ishibashi T, Sakamoto M, Mori M, Kondo C, Honji Y, Sun CR, Meng BY (1989). The complete sequence of the rice (*Oryza sativa*) chloroplast genome: intermolecular recombination between distinct tRNA genes accounts for a major plastid DNA inversion during the evolution of the cereals. Mol Gen Gene.

[CR15] Huesgen PF, Schuhmann H, Adamska I (2005). The family of Deg proteases in cyanobacteria and chloroplasts of higher plants. Physiol Plant.

[CR16] Inada H, Kusumi K, Nishimura M, Iba K (1996). Specific expression of the chloroplast gene for RNA polymerase (*rpoB*) at an early stage of leaf development in rice. Plant Cell Physiol.

[CR17] Itzhaki H, Naveh L, Lindahl M, Cook M, Adam Z (1998). Identification and characterization of DegP, a serine protease associated with the luminal side of the thylakoid membrane. J Biol Chem.

[CR18] Jarvis P, López-Juez E (2013). Biogenesis and homeostasis of chloroplasts and other plastids. Nat Rev Mol Cell Biol.

[CR19] Jiang Q, Mei J, Gong XD, Xu JL, Zhang JH, Teng S, Lin D, Dong Y (2014). Importance of the rice *TCD9* encoding alpha subunit of chaperonin protein60 (Cpn60α) for the chloroplast development during the early leaf stage. Plant Sci.

[CR20] Kato Y, Sakamoto W (2010). New insights into the types and function of proteases in plastids. Int Rev Cell Mol Biol.

[CR21] Kato Y, Sun X, Zhang LX, Sakamoto W (2012). Cooperative D1 degradation in the photosystem II repair mediated by chloroplastic proteases in Arabidopsis. Plant Physiol.

[CR22] Kolmar H, Waller P, Sauer R (1996). The DegP and DegQ periplasmic endoproteases of *Escherichia coli*: specificity for cleavage sites and substrate conformation. J Bacteriol.

[CR23] Krause GH, Weis E (1991). Chlorophyll fluorescence and photosynthesis: the basics. Annu Rev Plant Physiol Plant Mol Biol.

[CR24] Kurata N, Miyoshi K, Nonomura KI, Yamazaki Y, Ito Y (2005). Rice mutants and genes related to organ development, morphogenesis and physiological traits. Plant Cell Physiol.

[CR25] Kusumi K, Iba K (2014). Establishment of the chloroplast genetic system in rice during early leaf development and at low temperatures. Front Plant Sci.

[CR26] Kusumi K, Mizutani A, Nishimura M, Iba K (1997). A virescent gene *V1* determines the expression timing of plastid genes for transcription/translation apparatus during early leaf development in rice. Plant J.

[CR27] Kusumi K, Yara A, Mitsui N, Tozawa Y, Iba K (2004). Characterization of a rice nuclear-encoded plastid RNA polymerase gene *OsRpoTp*. Plant Cell Physiol.

[CR28] Kusumi K, Sakata C, Nakamura T, Kawasaki S, Yoshimura A, Iba K (2011). A plastid protein NUS1 is essential for build-up of the genetic system for early chloroplast development under cold stress conditions. Plant J.

[CR29] Leister D (2003). Chloroplast research in the genomic age. Trends Genet.

[CR30] Lipinska B, Sharma S, Georgopoulos C (1988). Sequence analysis and regulation of the HtrA gene of *Escherichia coli*: a sigma 32-independent mechanism of heat-inducible transcription. Nucleic Acids Res.

[CR31] Lipinska B, Zylicz M, Georgopoulos C (1990). The HtrA (DegP) protein, essential for *Escherichia coli* survival at high temperatures, is an endopeptidase. J Bacteriol.

[CR32] Liu W, Fu Y, Hu G, Si H, Zhu L, Wu C, Sun Z (2007). Identification and fine mapping of a thermo-sensitivechlorophyll deficient mutant in rice (*Oryza sativa* L.). Planta.

[CR33] Livak KJ, Schmittgen TD (2001). Analysis of relative gene expression data using real time quantitative PCR and the method. Methods.

[CR34] Lu HJ, Zhou XR, Gong ZX, Upadhyaya NM (2001). Generation of selectable marker-free transgenic rice using double right-border (DRB) binary vectors. Aust J Plant Physiol.

[CR35] Meurer J, Meierhoff K, Westhoff P (1996). Isolation of high-chlorophyll fluorescence mutants of *Arabidopsis thaliana* and their characterisation by spectroscopy, immunoblotting and northern hybridisation. Planta.

[CR36] Pendle AF, Clark GP, Boon R, Lewandowska D, Lam YW, Andersen J, Mann M, Lamond AI, Brown JW, Shaw PJ (2005). Proteomic analysis of the Arabidopsis nucleolus suggests novel nucleoli functions. Moll Boil Cell.

[CR37] Peng S, Garcia FV, Lasa RC, Classman KG (1993). Adjustment for specific leaf weight improves chlorophyll meter’s estimate of rice leaf nitrogen concentration. Agron J.

[CR38] Pfalz J, Pfannschmidt T (2013). Essential nucleoid proteins in early chloroplast development. Trends Plant Sci.

[CR39] Santis-Maciossek GD, Kofer W, Bock A, Schoch S, Maier RM, Wanner G, Rüdiger W, Koop HU (1999). Targeted disruption of the plastid RNA polymerase genes *rpoA*, B and C1: molecular biology, biochemistry and ultrastructure. Plant J.

[CR40] Schuhmann H, Huesgen PF, Gietl C, Adamska I (2008). The DEG15 serine protease cleaves peroxisomal targeting signal 2-containing proteins in *Arabidopsis thaliana*. Plant Physiol.

[CR41] Schuhmann H, Huesgen PF, Adamska I (2012). The family of Deg/HtrA proteases in plants. BMC Plant Biol.

[CR42] Strauch KL, Beckwith J (1988). An *Escherichia coli* mutation preventing degradation of abnormal periplasmic proteins. Proc Natl Acad Sci U S A.

[CR43] Sugimoto H, Kusumi K, Noguchi K, Yano M, Yoshimura A, Iba K (2007). The rice nuclear gene, *VIRESCENT2*, is essential for chloroplast development and encodes a novel type of guanylate kinase targeted to plastids and mitochondria. Plant J.

[CR44] Sun X, Fu T, Chen N, Guo J, Ma J, Zou M, Lu C, Zhang L (2010). The stromal chloroplast Deg7 protease participates in the repair of photosystem II after photoinhibition in Arabidopsis. Plant Physiol.

[CR45] Sun X, Ouyang M, Guo J, Ma J, Lu C, Adam Z, Zhang L (2010). The thylakoid protease Deg1 is involved in photosystem-II assembly in *Arabidopsis thaliana*. Plant J.

[CR46] Takeuchi R, Kimura S, Saotome A, Sakaguchi K (2007). Biochemical properties of a plastidial DNA polymerase of rice. Plant Mol Biol.

[CR47] Tanz SK, Castleden I, Hooper CM, Small I, Millar AH (2014). Using the SUBcellular database for Arabidopsis proteins to localize the Deg protease family. Front Plant Sci.

[CR48] Tripathi LP, Sowdhamini R (2006). Cross genome comparisons of serine proteases in Arabidopsis and rice. BMC Genomics.

[CR49] Turner FT, Jund MF (1991). Chlorophyll meter to predict nitrogen topdress requirement for semidwarf rice. Agron J.

[CR50] Vitha S, Mcandrew RS, Osteryoung KW (2001). *FtsZ* ring formation at the chloroplast division site in plants. J Cell Biol.

[CR51] Walsh NP, Alba BM, Bose B, Gross CA, Sauer RT (2003). OMP peptide signals initiate the envelope-stress response by activating DegS protease via relief of inhibition mediated by its PDZ domain. Cell.

[CR52] Yatou O, Cheng XY (1989). Temperature sensitive chlorophyll mutations. Rice Genet Newsletter.

[CR53] Yoo Y, Cho SH, Sugimoto H, Li J, Kusumi K, Koh H, Iba K, Paek N (2009). Rice *Virescent3* and *Stripe1* encoding the largeand small subunits of ribonucleotide reductase are required for chloroplast biogenesis during early leaf development. Plant Physiol.

[CR54] Yu J, Hu S, Wang J, Wong GK, Li S, Liu B, Deng Y, Dai L, Zhou Y, Zhang X, Cao M, Liu J, Sun J, Tang J, Chen Y, Huang X, Lin W, Ye C, Tong W, Cong L, Geng J, Han Y, Li L, Li W, Hu G, Huang X, Li W, Li J, Liu Z, Li L (2002). A draft sequence of the rice genome (*Oryza sativa* L. ssp. *indica*). Science.

