# Supplementary material for: The Rice TCM5 Gene Encoding a Novel Deg Protease Protein is Essential for Chloroplast Development under High Temperatures
Source: Rice (N Y). 2016 Mar 21;9:13. doi: 10.1186/s12284-016-0086-5 (PMC4801845; doi:10.1186/s12284-016-0086-5)
Supplement: Additional file 1: Figure S1. — The change of chlorophyll contents (SPAD values) in tcm5 and WT plants from the 1st (summer) to 12th weeks (heading date, autumn) after transplanting (2010, Shanghai, China). Figure S2. Comparative agronomic characteristics of tcm5 and wild type of rice plants grown under field condition (2010, Shanghai, China). PH, plant height; PN, panicle number per plant; GW: 1000-grain weight (g); GN, grains per panicle. Figure S3. Phylogenic analysis of rice Deg proteases inculding 10 Deg proteases and 6 Deg-like proteases proteins (Schuhmann et al., 2012) in the rice genome. Figure S4. Phylogenic analysis of TCM5 with all Arabidopsis Deg proteases. (PPTX 182 kb) [file 12284_2016_86_MOESM1_ESM.pptx]

## Slide 1
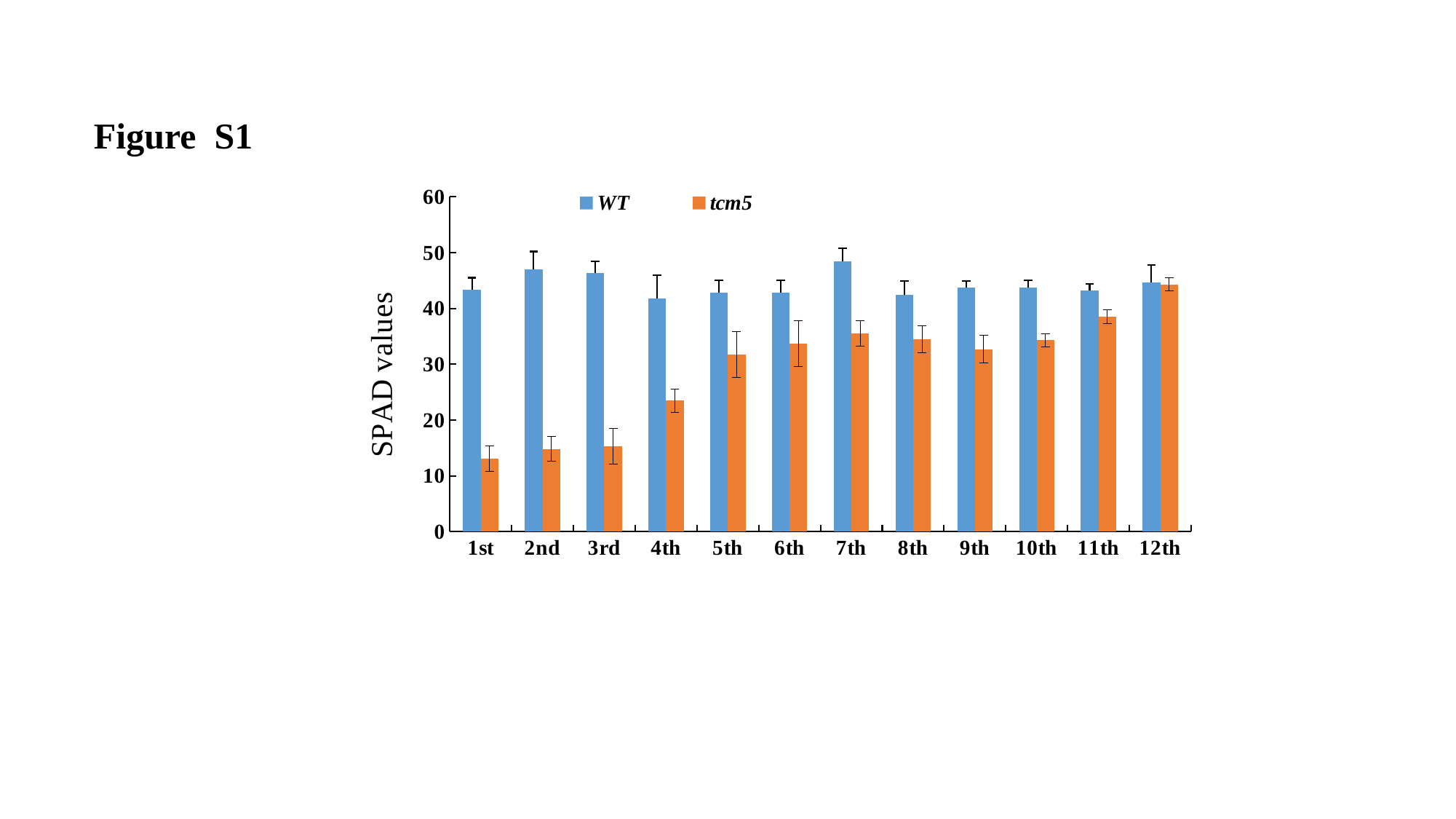

Figure S1
### Chart
| Category | WT | tcm5 |
|---|---|---|
| 1st | 43.3 | 13.1 |
| 2nd | 47.0 | 14.8 |
| 3rd | 46.3 | 15.3 |
| 4th | 41.8 | 23.5 |
| 5th | 42.8 | 31.7 |
| 6th | 42.8 | 33.7 |
| 7th | 48.4 | 35.5 |
| 8th | 42.4 | 34.47 |
| 9th | 43.7 | 32.7 |
| 10th | 43.8 | 34.300000000000004 |
| 11th | 43.2 | 38.5 |
| 12th | 44.7 | 44.3 |

## Slide 2
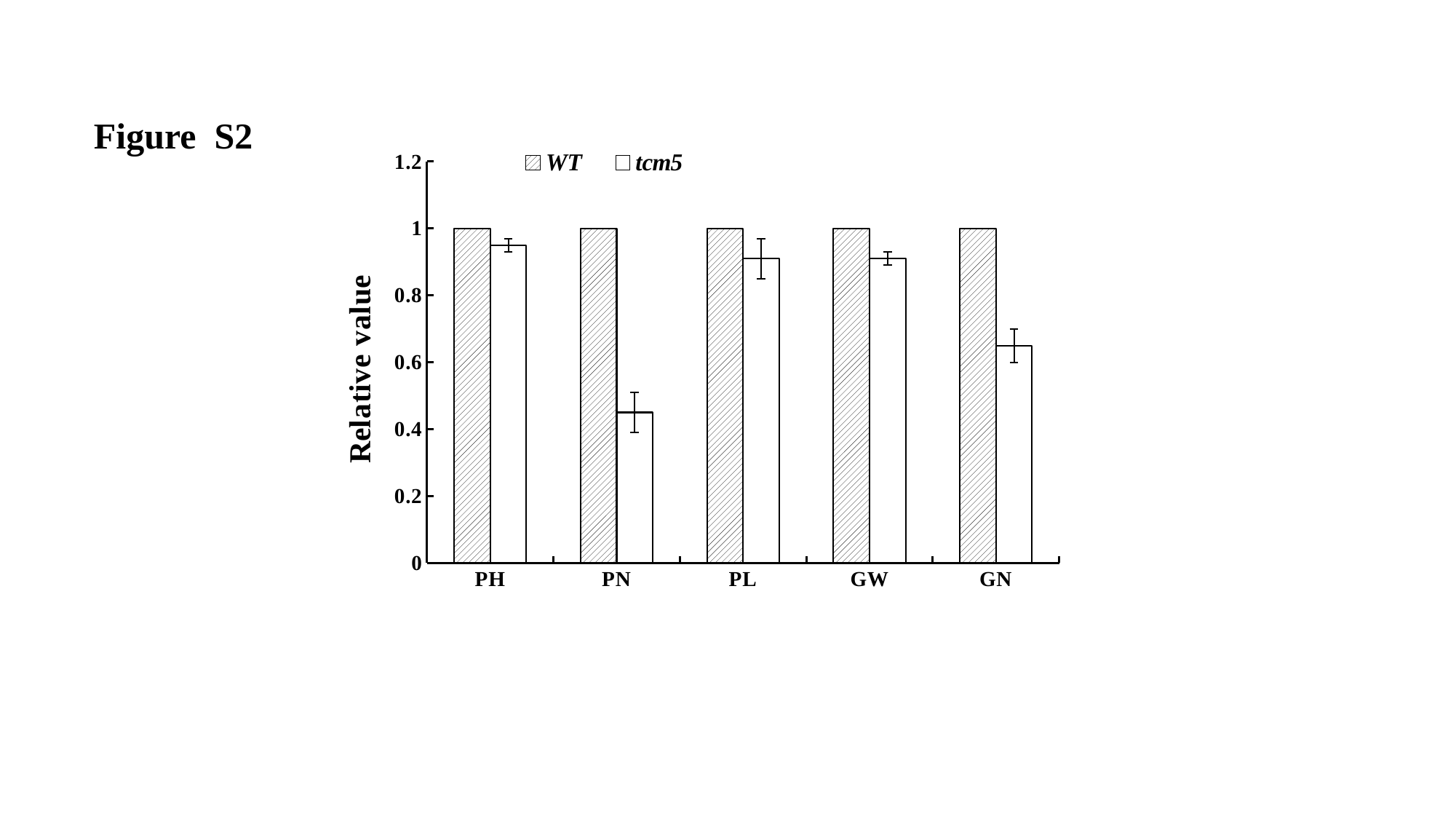

### Chart
| Category | WT | tcm5 |
|---|---|---|
| PH | 1.0 | 0.9500000000000006 |
| PN | 1.0 | 0.45 |
| PL | 1.0 | 0.91 |
| GW | 1.0 | 0.91 |
| GN | 1.0 | 0.650000000000001 |Figure S2

## Slide 3
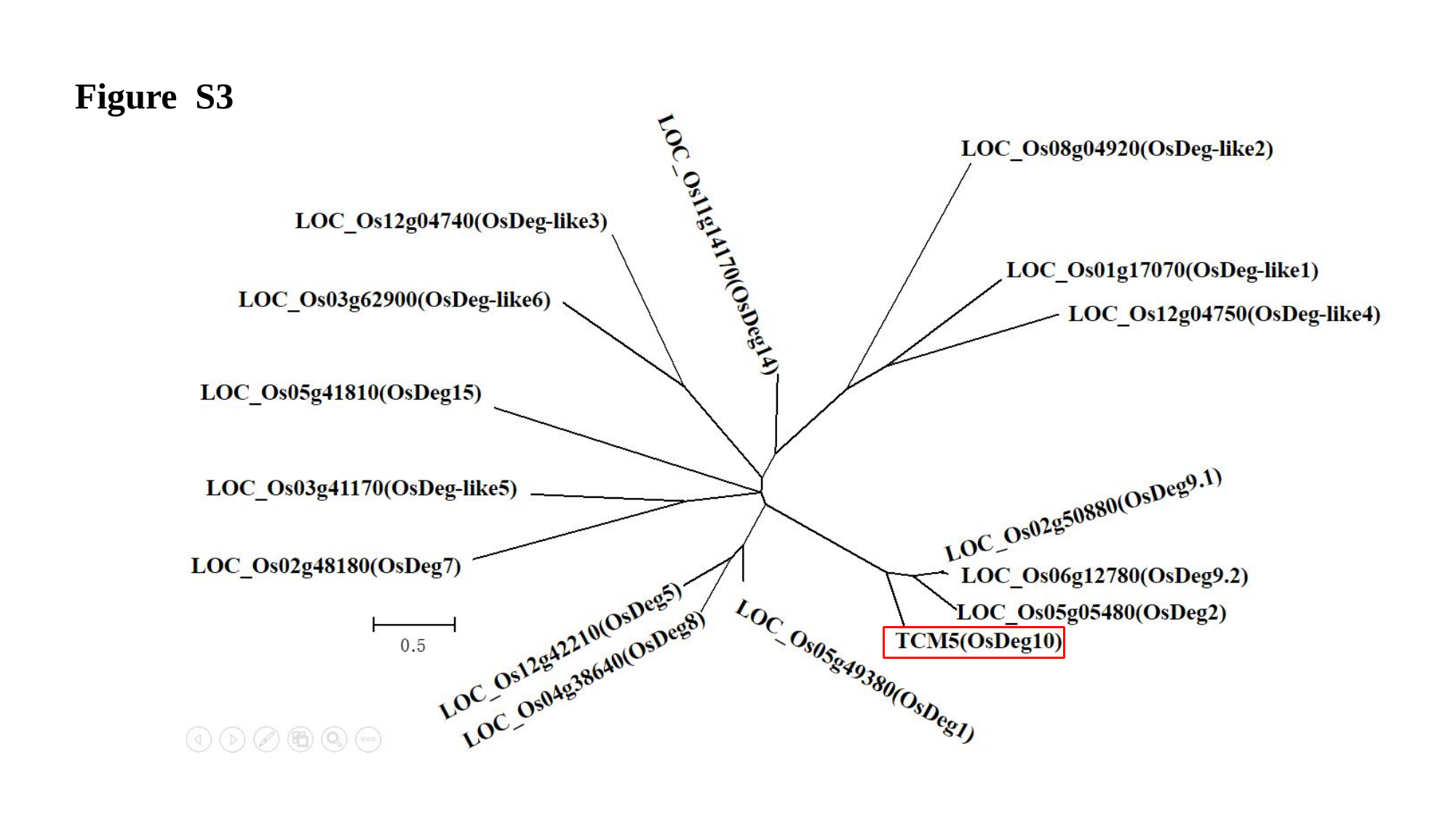

Figure S3

## Slide 4
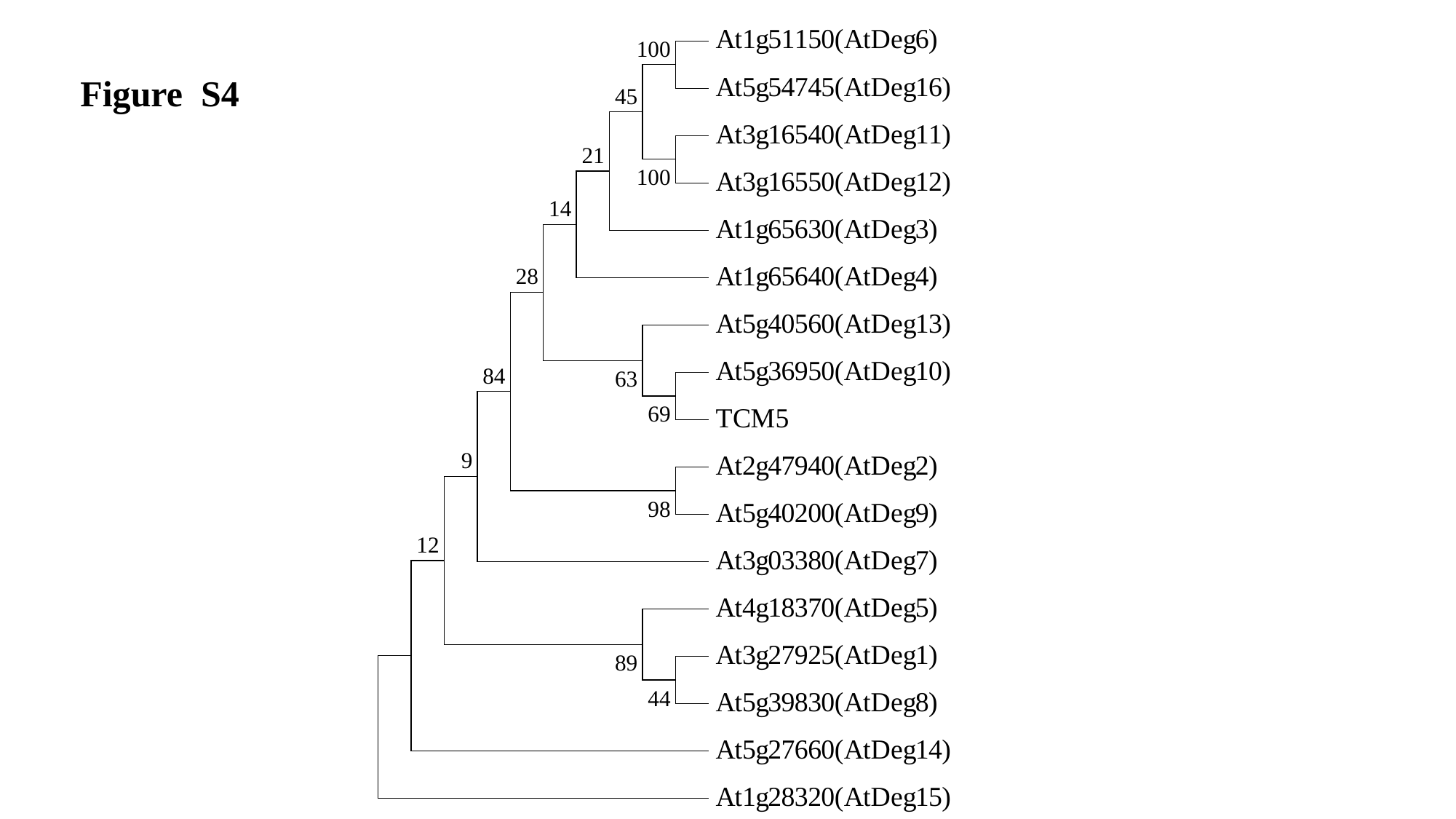

Figure S4
