# Supplementary material for: The Rice TCM5 Gene Encoding a Novel Deg Protease Protein is Essential for Chloroplast Development under High Temperatures
Source: Rice (N Y). 2016 Mar 21;9:13. doi: 10.1186/s12284-016-0086-5 (PMC4801845; doi:10.1186/s12284-016-0086-5)
Supplement: Additional file 2: Table S1. — The fluorescence induction parameters. Table S2. Genetic segregation analysis of tcm5 mutants in the F2 population. Table S3. The PCR-based molecular markers designed for fine mapping. Table S4. Markers designed for RT-PCR. (DOC 72 kb) [file 12284_2016_86_MOESM2_ESM.doc]

| **Table S1** The fluorescence induction parameters | | | | | |
| --- | --- | --- | --- | --- | --- |
|  | WT | |  | *tcm5* | |
|  | 20℃ | 32℃ |  | 20℃ | 32℃ |
| *Fv/Fm* | 0.791±0.04 | 0.787±0.03 |  | 0.783±0.02 | 0.365±0.02 |
| *ΦPS*II | 0.619±0.07 | 0.624±0.04 |  | 0.623±0.05 | 0.361±0.04 |

**Table S2** Genetic segregation analysis of *tcm5* mutants in the F_2_ population

| Cross | Observed number of F_2_ plants | | | χ2_(3:1)_ |
| --- | --- | --- | --- | --- |
|  | Total | Green | Albino |  |
| Pei'ai 64S/*tcm5* | 412 | 316 | 96 | 0.63 |

χ2_0.05_=3.84

| **Table S3** The PCR-based molecular markers designed for fine mapping | | | | |  |
| --- | --- | --- | --- | --- | --- |
| No. | Markers | Type | Forward sequence (5’ to 3’) | Reverse sequence (5’ to 3’) | Restriction site |
| 1 | RM2474 | SSR | GCTAGCTAGTACGGCGAGGA | CCGCAGCAGCTAAGCTAAAT |  |
| 2 | RM18692 | SSR | GCAGTTCGTTGTGGAGGAACACC | ATCGGCCACCCAAATCTTAATGG |  |
| 3 | RM4674 | SSR | AAGACCGAAATTAGACGGATGC | GGGTCTTATTACCTTCGTTTCAGG |  |
| 4 | RM18694 | SSR | CGCCTTCTCTGCATAGATCACC | GAAGAATCAGAAGCCGCTCTACG |  |
| 5 | P1 | InDel | GCGGCTATTGATTGCTCG | GATAGAGTCGTAGTCTCCCGTCC |  |
| 6 | P2 | CAPS | AGGACTGTCCGATCTTTGG | GAAGTCCAGCCCATTTACC | *TaqI* |
| 7 | P3 | CAPS | TTCTTCCTCCGCTTTTCCATCT | GTTATACCATGAGTACTCTGCAG | *AluI* |
| 8 | P4 | CAPS | TTTCAAGGGTTGTCGTCACTTCCGT | CCATGATTTGACAATGTGGCGCTAC | *TaqI* |

| **Table S4** Markers designed for realtime RT-PCR | | |
| --- | --- | --- |
| Genes | Forward primer | Reverse primer |
| *TCM5* | TCCGCTGCGATTCTTGCT | TCCTCTGCTCGTCCGTCAT |
| *rps7* | gccaaaatccattccaattc | ggagatgtacacgaggagattg |
| *rpl21* | AAGAAGAGGAGGCTGCGGT | GACATTGGCGCCTTTCAGC |
| *23SrRNA* | TGTGGGCGTTAGAGCATTGAG | CACTTGGCTACCCAGCGTTTA |
| *V1* | TAGTGGCTGTTGCCAGTGGA | TATCTAGTCTAACCACCAGC |
| *V2* | GAGGAGTTCCTCACGATGAT | AGCATCAATGATAGACTCC |
| *V3* | gttagatgcttcactacacag | gtaccattgccaacatggcaac |
| *LhcpII* | GAAGAAGATCAAGAACGGCC | TTGCCGGGGACGAAGTTGGT |
| *rbcS* | TCCGCTGAGTTTTGGCTATTT | GGACTTGAGCCCTGGAAGG |
| *FtsZ* | AAAGGACATAACCTTGCAAG | AGTTTTCCTATTGAACCGTG |
| *OsPOLP1* | ACCGGTGCTTTCAGGCTTGG | GCTGACTGATAATCACACG |
| *OsRpoTp* | AAGCAGACAGTGATGACATC | ATCACATGCATGCACCCAAA |
| *rpoB* | TTTGGTTTCGATGTGCA | TATGGTCTAATTCCGAGCGGT |
| *psaA* | GCGAGCAAATAAAACACCTTTC | GTACCAGCTTAACGTGGGGAG |
| *psbA* | CCCTCATTAGCAGATTCGTTTT | ATGATTGTATTCCAGGCAGAGC |
| *rbcL* | CTTGGCAGCATTCCGAGTAA | ACAACGGGCTCGATGTGATA |
| *rpoA* | GTGGAAGTGTGTTGAATCAA | TCTCTCTTGATCCGTAACTC |
| *CA01* | GATCCATACCCGATCGACAT | CGAGAGACATCCGGTAGAGC |
| *PORA* | TGTACTGGAGCTGGAACAACAA | GAGCACAGCAAAATCCTAGACG |
| *YGL1* | CAGTCTCCAATGGCCACCT | TGCTTTCATCAGTGGCTGGT |
| *cab1R* | AGATGGGTTTAGTGCGACGAG | TTTGGGATCGAGGGAGTATTT |
| *OsActin* | AGGAAGGCTGGAAGAGGACC | CGGGAAATTGTGAGGGACAT |
